# Genetic association between microRNA gene polymorphisms and polycystic ovary syndrome susceptibility: A systematic review and meta‐analysis

**DOI:** 10.1002/ijgo.70255

**Published:** 2025-06-10

**Authors:** Amaxsell Thiago Barros de Souza, Ketelly Leônara da Silva Torres, Ayane Cristine Alves Sarmento, Ariadne Sarynne Barbosa de Lima, Kleyton Santos de Medeiros, Deyse de Souza Dantas, Ricardo Ney Cobucci, Ana Katherine Gonçalves, Janaina Cristiana de Oliveira Crispim

**Affiliations:** ^1^ Postgraduate Program in Science Applied to Women's Health Federal University of Rio Grande Do Norte Natal Rio Grande do Norte Brazil; ^2^ Faculty of Pharmacy Federal University of Rio Grande Do Norte Natal Rio Grande do Norte Brazil; ^3^ Department of Clinical and Toxicological Analysis Federal University of Rio Grande Do Norte Natal Rio Grande do Norte Brazil; ^4^ Postgraduate Program in Health Sciences Federal University of Rio Grande Do Norte Natal Rio Grande do Norte Brazil; ^5^ Research and Innovation Teaching Institute Liga Contra o Câncer Natal Rio Grande do Norte Brazil; ^6^ Department of Molecular Biology Getúlio Sales Diagnostics Laboratory Natal Rio Grande do Norte Brazil; ^7^ Postgraduate Program of Biotechnology Potiguar University Natal Rio Grande do Norte Brazil; ^8^ Department of Obstetrics and Gynecology Federal University of Rio Grande Do Norte Natal Rio Grande do Norte Brazil

**Keywords:** meta‐analysis, microRNAs, polycystic ovary syndrome, polymorphism, single nucleotide, systematic review

## Abstract

**Background:**

Polycystic ovary syndrome (PCOS) is a prevalent endocrine disorder in women of reproductive age, associated with genetic and environmental factors, including microRNA (miRNA) gene polymorphisms.

**Objective:**

To evaluate the association between miRNA gene polymorphisms and PCOS.

**Search Strategy:**

PubMed, Embase, Web of Science, and Scopus databases were searched to September 2024, using MeSH terms including “MicroRNAs”, “Polymorphism, Single Nucleotide,” and “Polycystic Ovary Syndrome”.

**Selection Criteria:**

Case–control studies investigating the relationship between miRNA gene polymorphism and PCOS were included.

**Data Collection and Analysis:**

Two researchers collected the data independently. The risk of bias was assessed using the Newcastle‐Ottawa Scale (NOS). Data synthesis was performed using RevMan 5.4, and the strength of the evidence was evaluated using the grading of recommendations assessment, development, and evaluation (GRADE) approach.

**Main Results:**

Five case–control studies were included in the systematic review, encompassing 985 patients with PCOS and 1004 healthy controls. The meta‐analysis included data from 870 PCOS patients and 889 controls. The GG genotype of miR‐146a rs2910164 was significantly associated with a protective effect against PCOS, while the GC and CC genotypes were linked to increased PCOS risk. In contrast, the TT genotype of miR‐196a‐2 rs11614913 was associated with heightened PCOS susceptibility. However, the certainty of evidence supporting these associations was low, indicating that the true effects may differ from the observed estimates.

**Conclusion:**

miRNA polymorphisms, specifically the GG genotype of miR‐146a rs2910164 and the GC, CC, and TT genotypes of miR‐196a‐2 rs11614913 seem to be associated with an increased risk of PCOS, warranting further large‐scale studies to validate these associations.

## 1 INTRODUCTION

Polycystic ovary syndrome (PCOS) is the most common endocrine disorder among women of reproductive age and the leading cause of anovulatory infertility.^1^, ^2^, ^3^ It is characterized by chronic anovulation, hyperandrogenism, and menstrual irregularities, often accompanied by obesity, insulin resistance, and elevated luteinizing hormone (LH) levels.^4^ Women with PCOS are at an increased risk of developing insulin resistance, diabetes, cardiovascular complications, and cancers involving the ovaries, uterus, and breasts.^4^

The exact etiology of PCOS remains unclear, though it is believed to result from complex interactions between genetic and environmental factors contributing to its onset and progression.^5^ Genetic variations, particularly in specific loci, may predict an individual's susceptibility to PCOS. Research efforts are increasingly directed toward identifying potential candidate genes and single‐nucleotide polymorphisms (SNPs) associated with the condition to improve early diagnostic capabilities.

MicroRNAs (miRNAs), a class of small non‐coding RNAs, regulate approximately one‐third of human protein‐coding genes and play crucial roles in cellular proliferation, apoptosis, differentiation, immune response, and inflammation.^6^, ^7^ By acting at the post‐transcriptional level through translational repression and messenger RNA (mRNA) decay, miRNAs influence key cellular functions, including cell division, differentiation, and death.^8^, ^9^ In ovarian follicle development, including follicle growth and ovulation, miRNAs are particularly critical.^10^

Emerging evidence suggests that aberrant miRNA expression is associated with PCOS. Elevated miRNA levels have been observed in affected individuals, potentially distinguishing them from healthy controls.^11^, ^12^ These findings underscore the potential of miRNAs as non‐invasive biomarkers for early PCOS diagnosis due to their stability, resistance to nucleases, and ease of detection. Additionally, SNPs are common in the human genome and can influence disease risk. SNPs within miRNA genes, in particular, may alter miRNA expression or maturation, affecting their regulatory roles.^13^ Such polymorphisms can disrupt miRNA‐mediated cellular functions, potentially influencing PCOS development and progression.^14^

Although some studies have investigated the association of miRNA gene SNPs with gynecologic and reproductive conditions,^15^, ^16^ few have specifically explored their impact on PCOS. Systematic reviews play a critical role in synthesizing and evaluating the strength of existing evidence. Thus, this study aims to conduct a systematic review and meta‐analysis to assess the association between miRNA gene polymorphisms and PCOS risk, offering insights into potential biomarkers and therapeutic targets for the condition.

## 2 MATERIALS AND METHODS

### 2.1 Protocol and registration

This systematic review and meta‐analysis protocol was registered in the International Prospective Register of Systematic Reviews (PROSPERO) database (registration no.: CRD42024542478). The review followed the guidelines established by the Preferred Reporting Items for Systematic Reviews and Meta‐Analyses (PRISMA)^17^ and the Meta‐analysis of Observational Studies in Epidemiology (MOOSE)^18^ groups.

### 2.2 Eligibility criteria

To be eligible for inclusion, studies had to meet the following criteria: (i) Assess the association between miRNA gene polymorphisms and PCOS, (ii) provide genotype distributions necessary for calculating odds ratios (ORs) and 95% confidence intervals (CIs), and (iii) use a case–control design. The research question was structured according to the population, concept and context (PCC) format:

- Participants: women clinically diagnosed with PCOS by Rotterdam or National Institute of Health (NIH) criteria;
- Concept: miRNA gene polymorphisms increase or decrease PCOS risk;
- Context: case–control studies.

Abstracts, editorials, letters, review papers, and case reports; duplicate or overlapping publications; and studies lacking detailed genotype distribution data were excluded.

### 2.3 Data sources and search strategy

Databases searched included PubMed, Embase, Web of Science, and Scopus. A pilot search strategy was initially developed for PubMed and subsequently adapted for other databases, with systematic reviews and meta‐analyses on related topics included to identify additional studies. The search period was from inception up to September 2024, and no restrictions were imposed on publication year or language.

The MeSH (medical subject heading) terms and keywords included “MicroRNAs” AND “Polymorphism, Single Nucleotide” AND “Polycystic Ovary Syndrome”. The search strategies for each database are detailed in Table S1.

### 2.4 Selection process

Search results were imported into Rayyan software for duplicate removal, and an initial screening of 50 studies was conducted by two researchers (ATBS and KLST) to standardize selection criteria. Following this calibration, titles and abstracts of all identified studies were independently screened. Discrepancies were resolved through discussion or, if necessary, by a third researcher (JCOC). The same process was applied to the full‐text screening of selected studies.

### 2.5 Data extraction process

Data extraction was conducted using a form in Excel based on variables of interest. A pilot extraction was performed to ensure consistency, with two investigators (DSD and KSM) cross‐verifying the extracted data. Extracted information included the first author's name, publication year, study design, sample size, mean age, ethnicity, body mass index (BMI, calculated as weight in kilograms divided by the square of height in meters), genotype distributions, and Hardy–Weinberg equilibrium (HWE). Corresponding authors were contacted for missing data when necessary.

### 2.6 Assessment of risk of bias

Two researchers (ATBS and ACAS) independently assessed methodologic quality and risk of bias in the included studies using the Newcastle‐Ottawa Scale (NOS), a validated tool for evaluating nonrandomized studies in systematic reviews.^19^ Studies were assessed across three domains: selection, comparability, and exposure, with scores above seven points indicating high‐quality studies. Discrepancies were resolved by consensus or by a third reviewer (AKG).

### 2.7 Data analysis and reporting bias assessment

Meta‐analysis was conducted for miRNA polymorphisms examined in more than one study. Statistical analyses were performed using Review Manager (RevMan) software, version 5.4 (The Cochrane Collaboration, Copenhagen, Denmark). ORs with 95% CIs were calculated for each genotype to assess associations with PCOS. A Mantel–Haenszel fixed‐effects model was used if no significant heterogeneity was found, while a random‐effects model was applied if substantial heterogeneity was detected. Heterogeneity was assessed using the Chi‐square (*χ* ^2^) test and quantified with the *I* ^2^ statistic, with *I* ^2^ values of 25%, 50%, and 75% indicating low, moderate, and high heterogeneity, respectively. Subgroup analyses explored genotype‐specific effects, with forest plots displaying individual study estimates alongside pooled estimates. Egger's test was used to assess publication bias.

### 2.8 Certainty of evidence

The grading of recommendations, assessment, development, and evaluations (GRADE) method was employed to rate the certainty of evidence for each outcome as high, moderate, low, or very low, based on factors such as risk of bias, indirectness, inconsistency, imprecision, and publication bias.^20^

## 3 RESULTS

### 3.1 Study selection and characteristics

**FIGURE 1:**
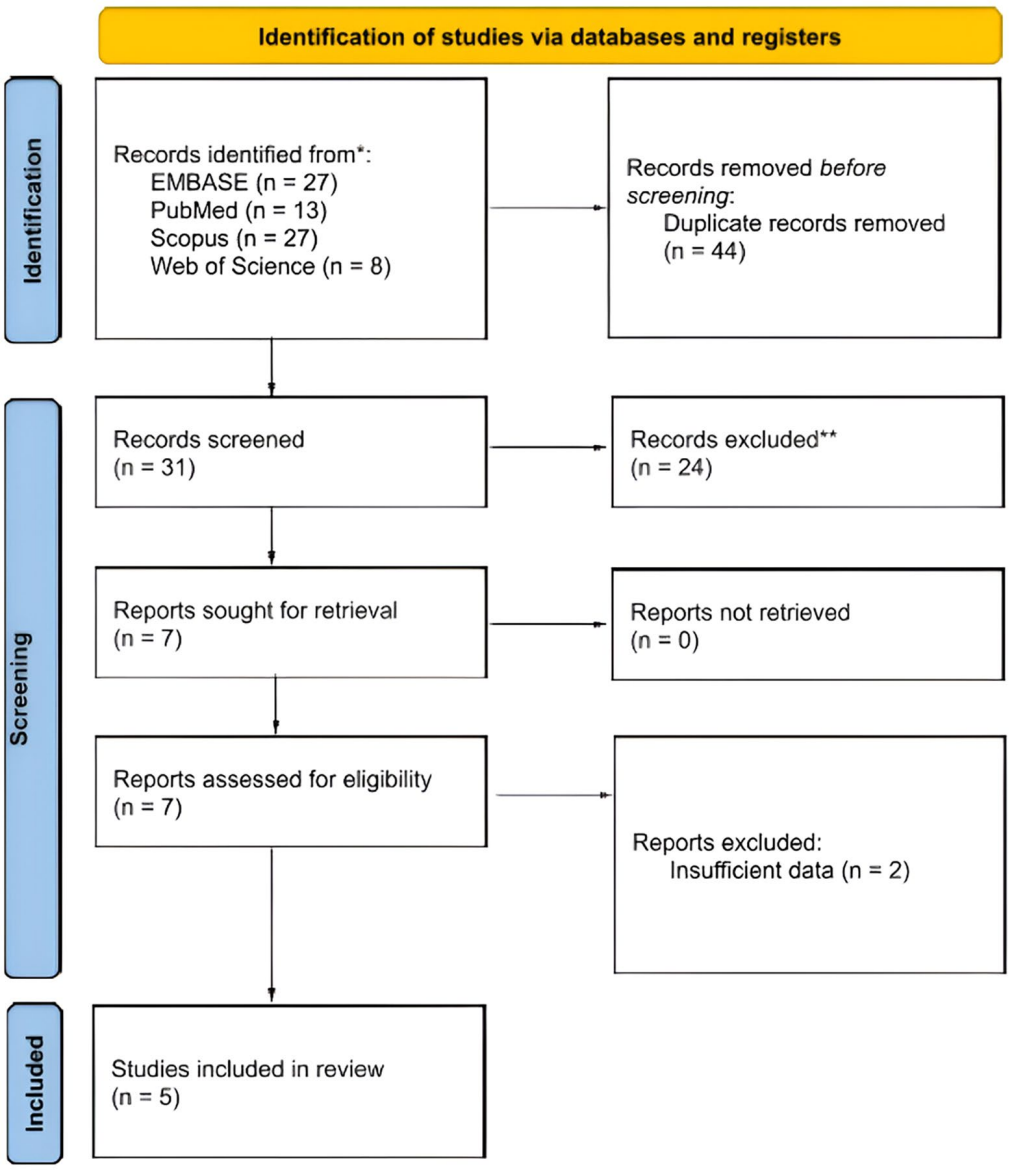
PRISMA flow chart of the study selection procedure.

The initial database search identified 75 studies. After accounting for additional sources from references and gray literature, we removed 44 duplicates, leaving 31 studies for title and abstract screening. Following this, 24 studies were excluded based on the criteria. Seven studies qualified for a full‐text review, and two were subsequently excluded due to insufficient data. Ultimately, five studies^21^, ^22^, ^23^, ^24^, ^25^ met the inclusion criteria and were included in the qualitative and quantitative synthesis, as illustrated in the PRISMA flow chart (Figure 1).

The selected studies, detailed in Table 1, were published between 2017 and 2022 and involved women from Iran^21^, ^22^, ^23^ of Persian ethnicity and Saudi Arabia^24^, ^25^ of Arabic ethnicity. Across these studies, 985 PCOS patients were included, with a mean age of 26.8–31.2 years. Most studies used the Rotterdam 2004 diagnostic criteria for PCOS,^22^, ^23^, ^24^, ^25^ while Ebrahimi et al.^21^ used the NIH criteria. The control group consisted of 1004 healthy women, with a mean age ranging from 27.0 to 28.6 years. In the case group, the mean BMI ranged from 23.23 to 27.79, while in the control group, it ranged from 22.92 to 26.1.

**TABLE 1:** Characteristics of included studies.

Author, year	Country	Age		Diagnostic criteria	Ethnicity/race	Sample size		BMI		microRNA involved	Polymorphisms studied
		Case	Control			Case	Control	Case	Control		
Ebrahimi et al. 2018^21^	Iran	26.8 ± 5.5	27.0 ± 4.38	National Institute of Health criteria	Persian	180	192	23.23 ± 3.6	22.92 ± 2.86	miR‐146a	miR‐146a rs2910164 (G>C)
Hossein et al. 2017^22^	Iran	31.2 ± 5.5	28.5 ± 5	Rotterdam criteria	Persian	205	205	26.5 ± 5	25.1 ± 4.6	miR‐146a miR‐222	miR‐146a rs2910164 (G>C) miR‐222 rs2858060 (G>C)
Li et al. 2022^23^	Iran	28.1 ± 0.31	28.6 ± 0.20	Rotterdam criteria	Persian	385	385	26.6 ± 5.7	26.1 ± 4.8	miR‐126 miR‐146a miR‐196a‐2 miR‐499	miR‐126 rs4636297 (G>A) miR‐146a rs2910164 (G>C) miR‐196a‐2 rs11614913 (C>T) miR‐499 rs3746444 (T>C)
Mir et al. 2022^24^	Saudi Arabia	27.59 ± 4.93	28.32 ± 4.12	Rotterdam criteria	Arabic	115	115	26.2 ± 2.52	23.71 ± 2.32	miR‐27a miR‐196a‐2 miR‐423	miR‐27a rs895819 (A>G) miR‐196a‐2 rs11614913 (C>T) miR‐423 rs6505162 (C>A)
Mir et al. 2022^25^	Saudi Arabia	27.89 ± 4.97	27.49 ± 4.29	Rotterdam criteria	Arabic	100	107	27.79 ± 4.82	25.71 ± 2.39	miR‐146a	miR‐146a rs2910164 (G>C)

*Note*: BMI, calculated as weight in kilograms divided by the square of height in meters.

Abbreviation: BMI, body mass index.

HWE was assessed for the control group in each study. Most polymorphisms met HWE criteria, except for those reported by Ebrahimi et al.^21^ and Hosseini et al.^22^ Across the five case–control studies, seven miRNA polymorphisms were evaluated for their potential association with PCOS. Each study focused on different miRNA polymorphisms: miR‐222 rs2858060 (G>C),^22^ miR‐126 rs4636297 (G>A),^23^ miR‐499 rs3746444 (T>C),^23^ miR‐27a rs895819 (A>G),^24^ miR‐423 rs6505162 (C>A),^24^ miR‐196a‐2 rs11614913 (T>G),^23^, ^24^ and miR‐146a rs2910164 (C>G).^21^, ^22^, ^23^, ^25^

### 3.2 Genotype distributions

The genotypic distribution of miRNA gene polymorphisms is shown in Table 2. For miR‐27a rs895819, Mir et al.^24^ reported that among PCOS cases, the GG genotype had the lowest frequency (*n* = 10), followed by the AA genotype (*n* = 40), while the GA genotype was the most frequent (*n* = 55). In controls, the AA genotype predominated (*n* = 60). For miR‐423 rs6505162, the CA genotype was the most prevalent in both the control group (*n* = 59) and the case group (*n* = 62). Li et al.^23^ analyzed miR‐126 rs4636297 and miR‐499 rs3746444, finding the heterozygous GA genotype to be most common among both cases (*n* = 184) and controls (*n* = 236), with GG being less frequent. In the case of miR‐499 rs3746444, the TT genotype was marginally more common among cases (*n* = 134) than controls (*n* = 188), while the heterozygous TC genotype was more frequent in controls. Hosseini et al.^22^ evaluated the miR‐222 rs2858060 polymorphism and reported that the GC genotype was most prevalent among both cases and controls, with frequencies of 134 in cases and 107 in controls.

**TABLE 2:** Distributions of microRNA gene polymorphism genotypes.

Gene polymorphism	Author, year	HWE	Case			Control		
			GG	GA	AA	GG	GA	AA
miR‐27a rs895819 (A>G)	Mir et al. 2022^24^	Yes	10	55	40	15	40	60
			**CC**	**CA**	**AA**	**CC**	**CA**	**AA**
miR‐423 rs6505162 (C>A)	Mir et al. 2022^24^	Yes	30	62	13	21	59	20
			**CC**	**CT**	**TT**	**CC**	**CT**	**TT**
miR‐196a‐2 rs11614913 (C>T)	Mir et al. 2022^24^	Yes	49	55	11	70	38	7
	Li et al. 2022^23^	Yes	147	168	70	159	178	48
			**GG**	**GC**	**CC**	**GG**	**GC**	**CC**
miR‐ 146a rs2910164 (C>G)	Ebrahimi et al. 2018^21^	No	113	58	9	154	34	4
	Hossein et al. 2017^22^	No	78	112	15	113	80	12
	Li et al. 2022^23^	Yes	163	168	54	244	119	22
	Mir et al. 2022^25^	Yes	27	43	30	49	40	18
			**GG**	**GC**	**CC**	**GG**	**GC**	**CC**
miR‐222 rs2858060 (G>C)	Hossein et al. 2017^22^	No	17	134	54	30	107	68
			**GG**	**GA**	**AA**	**GG**	**GA**	**AA**
miR‐126 rs4636297 (G>A)	Li et al. 2022^23^	Yes	184	169	32	236	135	14
			**TT**	**TC**	**CC**	**TT**	**TC**	**CC**
miR‐499 rs3746444 (T>C)	Li et al. 2022^23^	Yes	134	195	56	105	188	92

Abbreviation: HWE, Hardy–Weinberg equilibrium.

Both miR‐196a‐2 rs11614913 and miR‐146a rs2910164 were investigated in multiple studies. The CC genotype of miR‐196a‐2 rs11614913 was the most frequent among both cases and controls. Li et al.^23^ reported that 38 cases had the CC genotype compared to controls (*n* = 39), while the CT genotype was more frequent among cases (*n* = 49). miR‐146a rs2910164 was widely analyzed across studies.^21^, ^22^, ^23^, ^25^ The GC genotype was more common in cases (*n* = 381) than in controls (*n* = 273), whereas the GG genotype was more frequent in controls (*n* = 560) compared to cases (*n* = 381).

### 3.3 Quality assessment

The Newcastle‐Ottawa Scale was used to evaluate study quality, with most studies scoring seven out of nine (Table 3). The most common limitation observed was in the selection domain, where studies lacked representativeness in case selection.

**TABLE 3:** Quality assessment of included research papers using the Newcastle‐Ottawa Scale.

References	Selection				Comparability	Exposure			Overall quality score
	An adequate definition of cases	Representativeness of cases	Selection of controls	Definition of controls	Comparability of cases and controls based on design or analysis	Ascertainment of exposure	Same method for ascertainment for cases and controls	Non‐response rate	
Ebrahimi et al. 2018^21^	1	–	1	1	1	1	1	–	6
Hossein et al. 2017^22^	1	–	1	1	2	1	1	1	8
Li et al. 2022^23^	1	–	1	–	2	1	1	1	7
Mir et al. 2022^24^	1	–	1	1	2	1	1	1	8
Mir et al. 2022^25^	1	–	1	1	2	1	1	–	7

### 3.4 Meta‐analysis

We performed quantitative synthesis only for miRNAs analyzed in more than one study, focusing on miR‐196a‐2 rs11614913 (T>G) and miR‐146a rs2910164 (C>G). Two studies, totaling 500 PCOS cases and 500 controls, examined the association of miR‐196a‐2 rs11614913 with PCOS.^23^, ^24^ Notably, the TT genotype was significantly associated with an increased risk of PCOS (OR 1.57, 95% CI: 1.09–2.27, *I* ^2^ = 0%, *P* = 0.02), whereas the CC genotype was associated with a reduced risk of PCOS (OR 0.76, 95% CI: 0.59–0.98, *I* ^2^ = 75%, *P* = 0.03). In contrast, no statistically significant associations were found for the CT (OR 1.06, 95% CI: 0.82–1.36, *I* ^2^ = 82%, *P* = 0.66) genotypes, with high heterogeneity for these findings.

**FIGURE 2:**
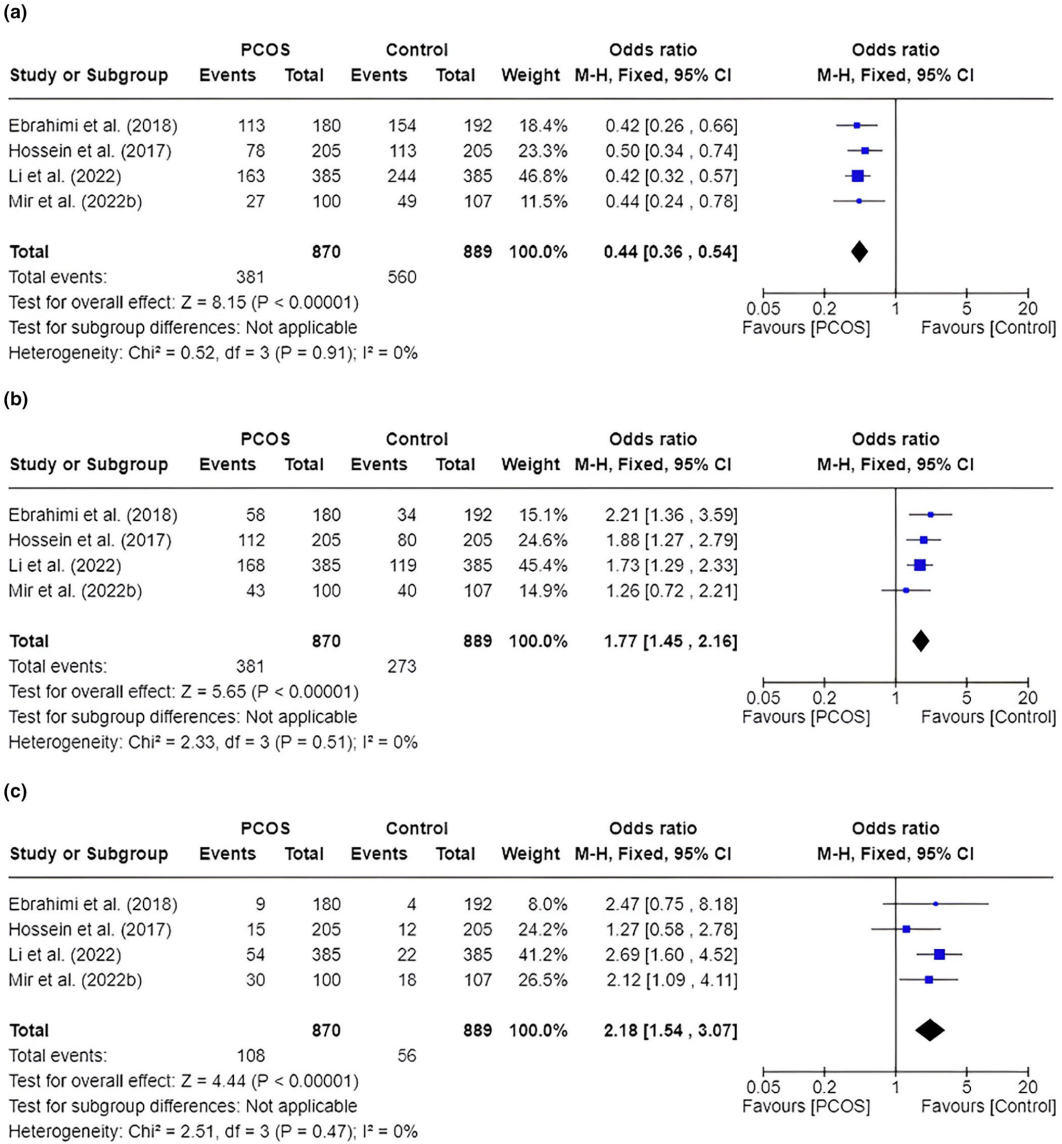
Forest plot of the association of miR‐146a rs2910164 (C>G) polymorphism with risk of polycystic ovary syndrome based on overall analysis. (a) GG genotype; (b) GC genotype; (c) CC genotype.

Our pooled analysis involving four studies^21^, ^22^, ^23^, ^25^ demonstrated a significant association between the miR‐146a rs2910164 polymorphism and PCOS risk. The pooled OR was 0.44 (95% CI: 0.36–0.54, *I* ^2^ = 0%, *P* < 0.00001), indicating a protective association between the GG genotype and PCOS (Figure 2). Analysis of the GC genotype yielded a pooled OR of 1.77 (95% CI: 1.45–2.16, *I* ^2^ = 0%, *P* < 0.00001), suggesting a 77% increased likelihood of PCOS in individuals with this genotype. The CC genotype also showed a significant association, with a pooled OR of 2.18 (95% CI: 1.54–3.07, *I* ^2^ = 0%, *P* < 0.00001), suggesting a two‐fold increased risk, with no heterogeneity reported.

### 3.5 Certainty of evidence

Table 4 summarizes the evidence certainty for the critical outcomes based on the GRADE approach. Both outcomes were rated as very low certainty due to biases, inconsistency, and publication bias, as indicated by the heterogeneity analysis.

**TABLE 4:** GRADE assessment.

Certainty assessment							
Outcomes	Participants (studies)	Risk of bias	Inconsistency	Indirectness	Imprecision	Publication bias	Overall certainty of evidence
miR‐196a‐2 rs11614913 (C>T)	500 cases 500 controls (2 non‐randomized studies)	Serious	Serious	Not serious	Not serious	Publication bias strongly suspected	⨁◯◯◯ Very low
miR‐146a rs2910164 (C>G)	870 cases 889 controls (4 non‐randomized studies)	Serious	Not serious	Not serious	Not serious	Publication bias strongly suspected	⨁◯◯◯ Very low

Abbreviations: CI, confidence interval; GRADE, grading of recommendations assessment, development, and evaluation; OR, odds ratio.

### 3.6 Reporting biases

Egger's linear regression test detected significant publication bias (*P* < 0.05).

## 4 DISCUSSION

This meta‐analysis reviewed case–control studies investigating the association of miRNA SNPs with PCOS. Among seven miRNA SNPs studied, meta‐analyses were conducted specifically on miR‐146a rs2910164 and miR‐196a‐2 rs11614913. Based on data from 870 PCOS patients and 889 healthy controls, the GG genotype of miR‐146a rs2910164 was significantly associated with a protective effect against PCOS, while the GC and CC genotypes were linked to increased risk. Conversely, the TT genotype of miR‐196a‐2 rs11614913 was associated with a heightened risk of PCOS. However, the evidence supporting these findings was of low certainty, indicating that the true effect might differ from the observed estimates.

The miR‐146a gene is critical in PCOS pathogenesis due to its role in regulating steroid hormone secretion, which influences levels of progesterone, estradiol, and testosterone.^26^ These hormonal imbalances contribute to the menstrual irregularity's characteristic of PCOS. Studies consistently report aberrant miR‐146a expression in women with PCOS, including findings by Long et al.^27^ demonstrating upregulated miR‐146a levels negatively correlated with serum testosterone. Elevated miR‐146a‐5p levels have been further associated with anovulation and insulin resistance.^28^, ^29^

We propose that the rs2910164 polymorphism in miR‐146a may influence disease susceptibility through its regulatory effects on gene expression. The G allele, associated with lower miR‐146a expression,^30^ may protect against PCOS by mitigating inflammatory and hormonal disruptions, whereas the C allele, linked to increased miR‐146a expression,^30^ may exacerbate disease risk by promoting TNF‐α production.^31^ Elevated TNF‐α levels contribute to insulin resistance, hyperandrogenism, and obesity, all of which are hallmarks of PCOS.^32^

Interestingly, the C allele has been linked to increased miR‐146a expression in ovarian granulosa cells, correlating with apoptosis—an important mechanism underlying defective follicular development in PCOS.^33^ These findings align with research in breast and ovarian cancer, where high miR‐146a expression is associated with the C allele.^34^ On the other hand, the GG genotype of miR‐146a is associated with an increased risk of ovarian cancer compared to the CC genotype in Chinese women,^35^ which is reinforced by studies showing that lower levels of miR‐146a in primary tumor tissue samples correlate with shorter progression‐free survival in ovarian cancer patients,^36^ considering their tumor suppressor effect highlighting the multifaceted role of miR‐146a in reproductive and metabolic health.

Additionally, the miR‐196a‐2 rs11614913 polymorphism affects the maturation of pre‐miRNA, with the T allele linked to lower synthesis of mature miR‐196a2.^37^ In our analysis, the TT genotype was significantly associated with increased PCOS risk. This SNP has also been implicated in conditions such as endometriosis,^38^ recurrent pregnancy loss,^39^ and endometrial and ovarian cancer,^40^ underscoring its relevance to female reproductive health.

The C allele, associated with higher miR‐196a2 expression, promotes cellular viability and migration in ovarian cells.^37^ Although the C allele has been linked to cardiovascular diseases^41^ and type 2 diabetes mellitus (T2DM),^42^ conditions closely related to PCOS. Our pooled analysis showed that the CC genotype was significantly associated with a reduced risk of PCOS. This finding suggests that, although the C allele has been linked to other metabolic disorders, it may play a protective role in PCOS. However, demographic factors such as ethnicity, age, and BMI may influence genotype distribution and modulate disease risk.

Furthermore, there are conflicting results regarding the role of the rs11614913 gene variation in female reproductive conditions. While the rs11614913 SNP has been linked to idiopathic recurrent abortion in Korean women,^43^ no such association was found in Iranian women.^44^ Similarly, combined genotypes, such as miR‐146a CG/miR‐196a2 TC, have shown protective effects against clinical phenotypes of premature ovarian failure^45^ and pre‐eclampsia,^46^ highlighting the nuanced role of miRNA variants in disease pathogenesis.

This is the first comprehensive meta‐analysis to assess the association between miRNA polymorphisms and PCOS risk. Nonetheless, several limitations should be acknowledged. Most included studies involved Persian and Arabian populations, which restricts the generalizability of the findings. Two studies had control groups deviating from Hardy–Weinberg equilibrium, suggesting possible genotyping errors or population stratification. The limited dataset hindered subgroup analyses by BMI, age, and clinical PCOS phenotypes. Inconsistent genotype reporting also precludes stratification by genetic models (e.g., dominant, recessive, codominant). Additionally, the small number of studies reduced the statistical power and the reliability of publication bias assessment using the Egger's test. According to the GRADE criteria, the overall certainty of evidence was low to very low, warranting cautious interpretation of the results.

## 5 CONCLUSIONS

This meta‐analysis provides evidence that the homozygous GG genotype of miR‐146a rs2910164 appears to confer a protective effect, while the GC and CC genotypes, as well as the TT genotype of miR‐196a‐2 rs11614913, increase PCOS risk. These findings suggest potential roles for miRNA polymorphisms as biomarkers. Further large‐scale multiethnic studies are necessary to confirm these associations and clarify the mechanisms by which miRNA SNPs contribute to PCOS pathogenesis, as current evidence is rated as low to very low certainty according to the GRADE.

## AUTHOR CONTRIBUTIONS

ATBS, KLST, ASBL, DSD and JCOC designed the study. ATBS and KLST searched the literature and extracted the data. ATBS and KSM performed statistical analyses. ATBS and ACAS realized the assessment of risk of bias. ATBS, KLST, ASBL, RNC and AKG. ATBS, RNC, AKG and JCOC critically revised successive drafts of the manuscript. All authors contributed to data analysis and manuscript revision, read and approved the publication of the final version.

## FUNDING INFORMATION

This research was funded by the Coordenação de Aperfeiçoamento de Pessoal de Nível Superior—Brazil (CAPES—Finance Code 001).

## CONFLICT OF INTEREST STATEMENT

The authors have no conflicts of interest.

## Supporting information


Table S1.

Table S2.

## Data Availability

Data sharing is not applicable to this article as no new data were created or analyzed in this study.

## References

[1] Azziz R , Kintziger K , Li R , et al. Recommendations for epidemiologic and phenotypic research in polycystic ovary syndrome: an androgen excess and PCOS society resource. Hum Reprod. 2019;34:2254‐2265. 31751476 10.1093/humrep/dez185

[2] Rebar RW , Keator CS . Polycystic ovary syndrome: consider the entire spectrum. Fertil Steril. 2024;121:934‐936. 38341057 10.1016/j.fertnstert.2024.02.008

[3] Alkhezi F , AlNemash N , AlMutairi J , et al. Prevalence of and risk factors associated with polycystic ovary syndrome among female university students of health sciences in a middle eastern country. Womens Health Rep (New Rochelle). 2024;5:579‐587. 39206018 10.1089/whr.2023.0176 PMC11347869

[4] Kim JJ , Choi YM , Hong MA , et al. FSH receptor gene p. Thr307Ala and p. Asn680Ser polymorphisms are associated with the risk of polycystic ovary syndrome. J Assist Reprod Genet. 2017;34:1087‐1093. 28547204 10.1007/s10815-017-0953-z PMC5533683

[5] Rosenfield RL , Ehrmann DA . The pathogenesis of polycystic ovary syndrome (PCOS): the hypothesis of PCOS as functional ovarian hyperandrogenism revisited. Endocr Rev. 2016;37:467‐520. 27459230 10.1210/er.2015-1104 PMC5045492

[6] Yu AM , Pan YZ . Noncoding microRNAs: small RNAs play a big role in regulation of ADME? Acta Pharm Sin B. 2012;2:93‐101. 32154096 10.1016/j.apsb.2012.02.011 PMC7061715

[7] Ha M , Kim VN . Regulation of microRNA biogenesis. Nat Rev Mol Cell Biol. 2014;15:509‐524. 25027649 10.1038/nrm3838

[8] Turchinovich A , Weiz L , Burwinkel B . Extracellular miRNAs: the mystery of their origin and function. Trends Biochem Sci. 2012;37:460‐465. 22944280 10.1016/j.tibs.2012.08.003

[9] O'Brien J , Hayder H , Zayed Y , Peng C . Overview of MicroRNA biogenesis, mechanisms of actions, and circulation. Front Endocrinol (Lausanne). 2018;9:402. 30123182 10.3389/fendo.2018.00402 PMC6085463

[10] Li Y , Fang Y , Liu Y , Yang X . MicroRNAs in ovarian function and disorders. J Ovarian Res. 2015;8:51. 26232057 10.1186/s13048-015-0162-2 PMC4522283

[11] Nasser JS , Altahoo N , Almosawi S , Alhermi A , Butler AE . The role of MicroRNA, long non‐coding RNA and circular RNA in the pathogenesis of polycystic ovary syndrome: a literature review. Int J Mol Sci. 2024;25:903. 38255975 10.3390/ijms25020903 PMC10815174

[12] Sørensen AE , Udesen PB , Wissing ML , Englund ALM , Dalgaard LT . MicroRNAs related to androgen metabolism and polycystic ovary syndrome. Chem Biol Interact. 2016;259:8‐16. 27270454 10.1016/j.cbi.2016.06.008

[13] Ryan BM , Robles AI , Harris CC . Genetic variation in microRNA networks: the implications for cancer research. Nat Rev Cancer. 2010;10:389‐402. 20495573 10.1038/nrc2867 PMC2950312

[14] Shi Y , Zhao H , Shi Y , et al. Genome‐wide association study identifies eight new risk loci for polycystic ovary syndrome. Nat Genet. 2012;44:1020‐1025. 22885925 10.1038/ng.2384

[15] Hu Y , Liu CM , Qi L , et al. Two common SNPs in pri‐miR‐125a alter the mature miRNA expression and associate with recurrent pregnancy loss in a Han‐Chinese population. RNA Biol. 2011;8:861‐872. 21788734 10.4161/rna.8.5.16034

[16] Dzay R , Mustafa S . Mir‐320b rs755613466 T>C and mir‐27a rs780199251 G>a polymorphisms and the risk of IVF failure in Kurdish women. Mol Biol Rep. 2020;47:1751‐1758. 32006196 10.1007/s11033-020-05266-0

[17] Page MJ , McKenzie JE , Bossuyt PM , et al. The PRISMA 2020 statement: an updated guideline for reporting systematic reviews. BMJ. 2021;372:n71. 33782057 10.1136/bmj.n71 PMC8005924

[18] Stroup DF , Berlin JA , Morton SC , et al. Meta‐analysis of observational studies in epidemiology: a proposal for reporting. Meta‐analysis of observational studies in epidemiology (MOOSE) group. JAMA. 2000;283:2008‐2012. 10789670 10.1001/jama.283.15.2008

[19] Stang A . Critical evaluation of the Newcastle‐Ottawa scale for the assessment of the quality of nonrandomized studies in meta‐analyses. Eur J Epidemiol. 2010;25:603‐605. 20652370 10.1007/s10654-010-9491-z

[20] Guyatt G , Oxman AD , Akl EA , et al. GRADE guidelines: 1. Introduction‐GRADE evidence profiles and summary of findings tables. J Clin Epidemiol. 2011;64:383‐394. 21195583 10.1016/j.jclinepi.2010.04.026

[21] Ebrahimi SO , Reiisi S , Parchami Barjui S . Increased risk of polycystic ovary syndrome (PCOS) associated with CC genotype of miR‐146a gene variation. Gynecol Endocrinol. 2018;34:793‐797. 29637801 10.1080/09513590.2018.1460341

[22] Hosseini AH , Kohan L , Aledavood A , Rostami S . Association of miR‐146a rs2910164 and miR‐222 rs2858060 polymorphisms with the risk of polycystic ovary syndrome in Iranian women: a case‐control study. Taiwan J Obstet Gynecol. 2017;56:652‐656. 29037553 10.1016/j.tjog.2017.08.014

[23] Li R , Yu Y , Jaafar SO , et al. Genetic variants miR‐126, miR‐146a, miR‐196a2, and miR‐499 in polycystic ovary syndrome. Br J Biomed Sci. 2022;79:10209. 35996522 10.3389/bjbs.2021.10209 PMC8915673

[24] Mir R , Saeedi NH , Jalal MM , et al. Clinical implications of Krüpple‐like transcription factor KLF‐14 and certain micro‐RNA (miR‐27a, miR‐196a2, miR‐423) gene variations as a risk factor in the genetic predisposition to PCOS. J Pers Med. 2022;12:586. 35455702 10.3390/jpm12040586 PMC9030665

[25] Mir R , Tayeb FJ , Barnawi J , et al. Biochemical characterization and molecular determination of estrogen receptor‐α (ESR1 PvuII‐rs2234693 T>C) and MiRNA‐146a (rs2910164 C>G) polymorphic gene variations and their association with the risk of polycystic ovary syndrome. Int J Environ Res Public Health. 2022;19:3114. 35270805 10.3390/ijerph19053114 PMC8910123

[26] Roth LW , McCallie B , Alvero R , Schoolcraft WB , Minjarez D , Katz‐Jaffe MG . Altered microRNA and gene expression in the follicular fluid of women with polycystic ovary syndrome. J Assist Reprod Genet. 2014;31:355‐362. 24390626 10.1007/s10815-013-0161-4 PMC3947080

[27] Long W , Zhao C , Ji C , et al. Characterization of serum microRNAs profile of PCOS and identification of novel non‐invasive biomarkers. Cell Physiol Biochem. 2014;33:1304‐1315. 24802714 10.1159/000358698

[28] Jiang X , Li J , Zhang B , et al. Differential expression profile of plasma exosomal microRNAs in women with polycystic ovary syndrome. Fertil Steril. 2021;115:782‐792. 33041053 10.1016/j.fertnstert.2020.08.019

[29] Roos J , Dahlhaus M , Funcke JB , et al. miR‐146a regulates insulin sensitivity via NPR3. Cell Mol Life Sci. 2021;78:2987‐3003. 33206203 10.1007/s00018-020-03699-1 PMC8004521

[30] Cho SH , Chung KW , Kim JO , et al. Association of miR‐146aC>G, miR‐149C>T, miR‐196a2T>C, and miR‐499A>G polymorphisms with risk of recurrent implantation failure in Korean women. Eur J Obstet Gynecol Reprod Biol. 2016;202:14‐19. 27156151 10.1016/j.ejogrb.2016.04.009

[31] Pauley KM , Satoh M , Chan AL , Bubb MR , Reeves WH , Chan EKL . Upregulated miR‐146a expression in peripheral blood mononuclear cells from rheumatoid arthritis patients. Arthritis Res Ther. 2008;10:R101. 18759964 10.1186/ar2493 PMC2575615

[32] Thathapudi S , Kodati V , Erukkambattu J , Katragadda A , Addepally U , Hasan Q . Tumor necrosis factor‐alpha and polycystic ovarian syndrome: a clinical, biochemical, and molecular genetic study. Genet Test Mol Biomarkers. 2014;18:605‐609. 25083576 10.1089/gtmb.2014.0151 PMC4150372

[33] Cho SH , An HJ , Kim KA , et al. Single nucleotide polymorphisms at miR‐146a/196a2 and their primary ovarian insufficiency‐related target gene regulation in granulosa cells. PLoS One. 2017;12:e0183479. 28841705 10.1371/journal.pone.0183479 PMC5571913

[34] Shen J , Ambrosone CB , DiCioccio RA , Odunsi K , Lele SB , Zhao H . A functional polymorphism in the miR‐146a gene and age of familial breast/ovarian cancer diagnosis. Carcinogenesis. 2008;29:1963‐1966. 18660546 10.1093/carcin/bgn172

[35] Sun XC , Zhang AC , Tong LL , et al. miR‐146a and miR‐196a2 polymorphisms in ovarian cancer risk. Genet Mol Res. 2016;15:gmr.1503468. 10.4238/gmr.15038468 27706635

[36] Wilczyński M , Żytko E , Szymańska B , et al. Expression of miR‐146a in patients with ovarian cancer and its clinical significance. Oncol Lett. 2017;14:3207‐3214. 28927067 10.3892/ol.2017.6477 PMC5588008

[37] Song ZS , Wu Y , Zhao HG , et al. Association between the rs11614913 variant of miRNA‐196a‐2 and the risk of epithelial ovarian cancer. Oncol Lett. 2016;11:194‐200. 26870188 10.3892/ol.2015.3877 PMC4727068

[38] Farsimadan M , Ismail Haje M , Khudhur Mawlood C , et al. MicroRNA variants in endometriosis and its severity. Br J Biomed Sci. 2021;78:206‐210. 33583336 10.1080/09674845.2021.1889157

[39] Srivastava P , Bamba C , Chopra S , Mandal K . Role of miRNA polymorphism in recurrent pregnancy loss: a systematic review and meta‐analysis. Biomark Med. 2022;16:101‐115. 35026953 10.2217/bmm-2021-0568

[40] Liu X , Xu B , Li S , et al. Association of SNPs in miR‐146a, miR‐196a2, and miR‐499 with the risk of endometrial/ovarian cancer. Acta Biochim Biophys Sin (Shanghai). 2015;47:564‐566. 26008204 10.1093/abbs/gmv042

[41] Sung JH , Kim SH , Yang WI , et al. miRNA polymorphisms (miR‐146a, miR‐149, miR‐196a2 and miR‐499) are associated with the risk of coronary artery disease. Mol Med Rep. 2016;14:2328‐2342. 27430349 10.3892/mmr.2016.5495

[42] Khan MS , Rahman B , Haq TU , et al. Deciphering the variants located in the MIR196A2, MIR146A, and MIR423 with Type‐2 diabetes mellitus in Pakistani population. Genes (Basel). 2021;12:664. 33925232 10.3390/genes12050664 PMC8146332

[43] Jeon YJ , Choi YS , Rah H , et al. Association study of microRNA polymorphisms with risk of idiopathic recurrent spontaneous abortion in Korean women. Gene. 2012;494:168‐173. 22222140 10.1016/j.gene.2011.12.026

[44] Fazli M , Ghorbian S . Association study of non‐coding RNA miR‐499 and miR196a2 gene polymorphisms with the risk of idiopathic recurrent pregnancy loss. Gene Cell Tissue. 2018;5:e67253.

[45] Rah H , Jeon YJ , Shim SH , et al. Association of miR‐146aC>G, miR‐196a2T>C, and miR‐499A>G polymorphisms with risk of premature ovarian failure in Korean women. Reprod Sci. 2013;20:60‐68. 22872486 10.1177/1933719112450341

[46] Asadi‐Tarani M , Saravani M , Teimoori B , Ghasemi M , Salimi S . The relationships between maternal and placental polymorphisms of miR‐196a2 and miRNA‐499 genes and preeclampsia. Br J Biomed Sci. 2020;77:191‐195. 32686997 10.1080/09674845.2020.1769331

