# Supplementary material for: Genetic association between microRNA gene polymorphisms and polycystic ovary syndrome susceptibility: A systematic review and meta‐analysis
Source: Int J Gynaecol Obstet. 2025 Jun 10;171(2):629–38. doi: 10.1002/ijgo.70255 (PMC12553106; doi:10.1002/ijgo.70255)
Supplement: Supplementary file 1 — Table S1. [file IJGO-171-629-s001.docx]

**Table S1:** Search strategy.

| **PubMed** | **Total Number of Papers** |
| --- | --- |
| ("microrna s"[All Fields] OR "micrornas"[MeSH Terms] OR "micrornas"[All Fields] OR "microrna"[All Fields] OR ("micrornas"[MeSH Terms] OR "micrornas"[All Fields] OR ("micro"[All Fields] AND "rna"[All Fields]) OR "micro rna"[All Fields]) OR ("micrornas"[MeSH Terms] OR "micrornas"[All Fields] OR ("rna"[All Fields] AND "micro"[All Fields]) OR "rna micro"[All Fields]) OR ("microrna s"[All Fields] OR "micrornas"[MeSH Terms] OR "micrornas"[All Fields] OR "microrna"[All Fields]) OR ("micrornas"[MeSH Terms] OR "micrornas"[All Fields] OR "mirna"[All Fields] OR "mirnas"[All Fields] OR "mirna s"[All Fields]) OR ("micrornas"[MeSH Terms] OR "micrornas"[All Fields] OR "mirna"[All Fields] OR "mirnas"[All Fields] OR "mirna s"[All Fields])) |  |
| ("polymorphism, single nucleotide"[MeSH Terms] OR ("polymorphism"[All Fields] AND "single"[All Fields] AND "nucleotide"[All Fields]) OR "single nucleotide polymorphism"[All Fields] OR "polymorphism single nucleotide"[All Fields] OR ("polymorphism, single nucleotide"[MeSH Terms] OR ("polymorphism"[All Fields] AND "single"[All Fields] AND "nucleotide"[All Fields]) OR "single nucleotide polymorphism"[All Fields] OR ("nucleotide"[All Fields] AND "polymorphism"[All Fields] AND "single"[All Fields]) OR "nucleotide polymorphism single"[All Fields]) OR ("polymorphism, single nucleotide"[MeSH Terms] OR ("polymorphism"[All Fields] AND "single"[All Fields] AND "nucleotide"[All Fields]) OR "single nucleotide polymorphism"[All Fields] OR ("nucleotide"[All Fields] AND "polymorphisms"[All Fields] AND "single"[All Fields]) OR "nucleotide polymorphisms single"[All Fields]) OR ("polymorphism, single nucleotide"[MeSH Terms] OR ("polymorphism"[All Fields] AND "single"[All Fields] AND "nucleotide"[All Fields]) OR "single nucleotide polymorphism"[All Fields] OR ("polymorphisms"[All Fields] AND "single"[All Fields] AND "nucleotide"[All Fields]) OR "polymorphisms single nucleotide"[All Fields]) OR ("polymorphism, single nucleotide"[MeSH Terms] OR ("polymorphism"[All Fields] AND "single"[All Fields] AND "nucleotide"[All Fields]) OR "single nucleotide polymorphism"[All Fields] OR ("single"[All Fields] AND "nucleotide"[All Fields] AND "polymorphisms"[All Fields]) OR "single nucleotide polymorphisms"[All Fields]) OR ("polymorphism, single nucleotide"[MeSH Terms] OR ("polymorphism"[All Fields] AND "single"[All Fields] AND "nucleotide"[All Fields]) OR "single nucleotide polymorphism"[All Fields] OR ("single"[All Fields] AND "nucleotide"[All Fields] AND "polymorphism"[All Fields])) OR ("polymorphism, single nucleotide"[MeSH Terms] OR ("polymorphism"[All Fields] AND "single"[All Fields] AND "nucleotide"[All Fields]) OR "single nucleotide polymorphism"[All Fields] OR "snps"[All Fields])) |  |
| ("polycystic ovary syndrome"[MeSH Terms] OR ("polycystic"[All Fields] AND "ovary"[All Fields] AND "syndrome"[All Fields]) OR "polycystic ovary syndrome"[All Fields] OR ("polycystic ovary syndrome"[MeSH Terms] OR ("polycystic"[All Fields] AND "ovary"[All Fields] AND "syndrome"[All Fields]) OR "polycystic ovary syndrome"[All Fields] OR ("ovary"[All Fields] AND "syndrome"[All Fields] AND "polycystic"[All Fields]) OR "ovary syndrome polycystic"[All Fields]) OR ("polycystic ovary syndrome"[MeSH Terms] OR ("polycystic"[All Fields] AND "ovary"[All Fields] AND "syndrome"[All Fields]) OR "polycystic ovary syndrome"[All Fields] OR ("syndrome"[All Fields] AND "polycystic"[All Fields] AND "ovary"[All Fields]) OR "syndrome polycystic ovary"[All Fields]) OR ("polycystic ovary syndrome"[MeSH Terms] OR ("polycystic"[All Fields] AND "ovary"[All Fields] AND "syndrome"[All Fields]) OR "polycystic ovary syndrome"[All Fields] OR ("polycystic"[All Fields] AND "ovarian"[All Fields] AND "syndrome"[All Fields]) OR "polycystic ovarian syndrome"[All Fields]) OR ("polycystic ovary syndrome"[MeSH Terms] OR ("polycystic"[All Fields] AND "ovary"[All Fields] AND "syndrome"[All Fields]) OR "polycystic ovary syndrome"[All Fields] OR ("ovarian"[All Fields] AND "syndrome"[All Fields] AND "polycystic"[All Fields]) OR "ovarian syndrome polycystic"[All Fields]) OR ("polycystic ovary syndrome"[MeSH Terms] OR ("polycystic"[All Fields] AND "ovary"[All Fields] AND "syndrome"[All Fields]) OR "polycystic ovary syndrome"[All Fields] OR "polycystic ovary syndrome 1"[All Fields]) OR ("polycystic ovary syndrome"[MeSH Terms] OR ("polycystic"[All Fields] AND "ovary"[All Fields] AND "syndrome"[All Fields]) OR "polycystic ovary syndrome"[All Fields] OR ("sclerocystic"[All Fields] AND "ovarian"[All Fields] AND "degeneration"[All Fields]) OR "sclerocystic ovarian degeneration"[All Fields]) OR ("polycystic ovary syndrome"[MeSH Terms] OR ("polycystic"[All Fields] AND "ovary"[All Fields] AND "syndrome"[All Fields]) OR "polycystic ovary syndrome"[All Fields] OR ("ovarian"[All Fields] AND "degeneration"[All Fields] AND "sclerocystic"[All Fields])) OR ("polycystic ovary syndrome"[MeSH Terms] OR ("polycystic"[All Fields] AND "ovary"[All Fields] AND "syndrome"[All Fields]) OR "polycystic ovary syndrome"[All Fields] OR ("sclerocystic"[All Fields] AND "ovary"[All Fields] AND "syndrome"[All Fields]) OR "sclerocystic ovary syndrome"[All Fields]) OR ("polycystic ovary syndrome"[MeSH Terms] OR ("polycystic"[All Fields] AND "ovary"[All Fields] AND "syndrome"[All Fields]) OR "polycystic ovary syndrome"[All Fields] OR ("stein"[All Fields] AND "leventhal"[All Fields] AND "syndrome"[All Fields]) OR "stein leventhal syndrome"[All Fields]) OR ("polycystic ovary syndrome"[MeSH Terms] OR ("polycystic"[All Fields] AND "ovary"[All Fields] AND "syndrome"[All Fields]) OR "polycystic ovary syndrome"[All Fields] OR ("stein"[All Fields] AND "leventhal"[All Fields] AND "syndrome"[All Fields]) OR "stein leventhal syndrome"[All Fields]) OR (("syndrom"[All Fields] OR "syndromal"[All Fields] OR "syndromally"[All Fields] OR "syndrome"[MeSH Terms] OR "syndrome"[All Fields] OR "syndromes"[All Fields] OR "syndrome s"[All Fields] OR "syndromic"[All Fields] OR "syndroms"[All Fields]) AND "Stein-Leventhal"[All Fields]) OR ("polycystic ovary syndrome"[MeSH Terms] OR ("polycystic"[All Fields] AND "ovary"[All Fields] AND "syndrome"[All Fields]) OR "polycystic ovary syndrome"[All Fields] OR ("sclerocystic"[All Fields] AND "ovaries"[All Fields]) OR "sclerocystic ovaries"[All Fields]) OR ("polycystic ovary syndrome"[MeSH Terms] OR ("polycystic"[All Fields] AND "ovary"[All Fields] AND "syndrome"[All Fields]) OR "polycystic ovary syndrome"[All Fields] OR ("ovary"[All Fields] AND "sclerocystic"[All Fields])) OR ("polycystic ovary syndrome"[MeSH Terms] OR ("polycystic"[All Fields] AND "ovary"[All Fields] AND "syndrome"[All Fields]) OR "polycystic ovary syndrome"[All Fields] OR ("sclerocystic"[All Fields] AND "ovary"[All Fields]) OR "sclerocystic ovary"[All Fields])) |  |
| ‎ #1 AND #2 AND #3 | 13 |
| **Web of Sciences** | |
| ((((ALL=(microRNA)) OR ALL=(micro RNA)) OR ALL=(microRNAs)) OR ALL=(miRNA)) OR ALL=(miRNAs) |  |
| ((((ALL=(single nucleotide polymorphism)) OR ALL=(polymorphism, single nucleotide)) OR ALL=(single nucleotide variant)) OR ALL=(single nucleotide variation)) OR ALL=(single nucleotide polymorphism) |  |
| (((((((((((((((ALL=(ovary polycystic disease)) OR ALL=(cystic ovary)) OR ALL=(micropolycystic ovary)) OR ALL=(multiple follicle cyst)) OR ALL=(ovary polycystic syndrome)) OR ALL=(ovary, micropolycystic)) OR ALL=(ovary, polycystic)) OR ALL=(polycystic ovarian disease)) OR ALL=(polycystic ovary)) OR ALL=(polycystic ovary disease)) OR ALL=(polycystic ovary syndrome)) OR ALL=(stein cohen leventhal syndrome)) OR ALL=(stein leventhal disease)) OR ALL=(Stein Leventhal syndrome)) OR ALL=(syndrome stein leventhal)) OR ALL=(ovary polycystic disease) |  |
| ‎ #1 AND #2 AND #3 | 8 |
| **SCOPUS** | |
| TITLE-ABS-KEY ( "microRNA" OR "micro RNA" OR "microRNAs" OR "miRNA" OR "miRNAs" ) |  |
| TITLE-ABS-KEY ( "ovary polycystic disease" OR "cystic ovary" OR "micropolycystic ovary" OR "multiple follicle cyst" OR "ovary polycystic syndrome" OR "ovary, micropolycystic" OR "ovary, polycystic" OR "polycystic ovarian disease" OR "polycystic ovary" OR "polycystic ovary disease" OR "polycystic ovary syndrome" OR "stein cohen leventhal syndrome" OR "stein leventhal disease" OR "Stein Leventhal syndrome" OR "syndrome stein leventhal" OR "ovary polycystic disease" ) |  |
| TITLE-ABS-KEY ( "single nucleotide polymorphism" OR "polymorphism, single nucleotide" OR "single nucleotide variant" OR "single nucleotide variation" OR "single nucleotide polymorphism" ) |  |
| #1 AND #2 AND #3 | 27 |
| **Embase** | |
| 'microRNA' OR 'micro RNA' OR 'microRNAs' OR 'miRNA' OR 'miRNAs' |  |
| 'single nucleotide polymorphism' OR 'polymorphism, single nucleotide' OR 'single nucleotide variant' OR 'single nucleotide variation' OR 'single nucleotide polymorphism' |  |
| 'ovary polycystic disease' OR 'cystic ovary' OR 'micropolycystic ovary' OR 'multiple follicle cyst' OR 'ovary polycystic syndrome' OR 'ovary, micropolycystic' OR 'ovary, polycystic' OR 'polycystic ovarian disease' OR 'polycystic ovary' OR 'polycystic ovary disease' OR 'polycystic ovary syndrome' OR 'stein cohen leventhal syndrome' OR 'stein leventhal disease' OR 'Stein Leventhal syndrome' OR 'syndrome stein leventhal' OR 'ovary polycystic disease' |  |
| ‎#1 AND #2 AND #3 | 27 |
